# Solitary Large Hepatocellular Carcinoma: Staging and Treatment Strategy

**DOI:** 10.1371/journal.pone.0155588

**Published:** 2016-05-13

**Authors:** Po-Hong Liu, Chien-Wei Su, Chia-Yang Hsu, Cheng-Yuan Hsia, Yun-Hsuan Lee, Yi-Hsiang Huang, Rheun-Chuan Lee, Han-Chieh Lin, Teh-Ia Huo

**Affiliations:** 1 Department of Medicine, Taipei Veterans General Hospital, Taipei, Taiwan; 2 Department of Surgery, Taipei Veterans General Hospital, Taipei, Taiwan; 3 Department of Radiology, Taipei Veterans General Hospital, Taipei, Taiwan; 4 Faculty of Medicine, National Yang-Ming University School of Medicine, Taipei, Taiwan; 5 Institute of Clinical Medicine, National Yang-Ming University School of Medicine, Taipei, Taiwan; 6 Institute of Pharmacology, National Yang-Ming University School of Medicine, Taipei, Taiwan; 7 Department of Internal Medicine, University of Nevada School of Medicine, Reno, Nevada, United States of America; Kaohsiung Chang Gung Memorial Hospital, TAIWAN

## Abstract

**Background & Aims:**

Controversies exist on staging and management of solitary large (>5 cm) hepatocellular carcinoma (HCC). This study aims to evaluate the impact of tumor size on Barcelona Clinic Liver Cancer (BCLC) staging and treatment strategy.

**Methods:**

BCLC stage A and B patients were included and re-classified as single tumor 2–5 cm or up to 3 tumors ≤3 cm (group A; n = 657), single tumor >5 cm (group SL; n = 224), and multiple tumors >3 cm (group B; n = 351). Alternatively, 240 and 229 patients with solitary large HCC regardless of tumor stage received surgical resection (SR) and transarterial chemoembolization (TACE), respectively. The propensity score analysis identified 156 pairs of patients from each treatment arm for survival comparison.

**Results:**

The survival was significantly higher for group A but was comparable between group SL and group B patients. Of patients with solitary large HCC, the 1-, 3- and 5-year survival rates were 88% *versus* 74%, 76% *versus* 44%, and 63% *versus* 35% between SR and TACE group, respectively (*p*<0.001). When baseline demographics were adjusted in the propensity model, the respective 1-, 3- and 5-year survival rates were 87% *versus* 79%, 76% *versus* 46%, and 61% *versus* 36% (*p*<0.001). The Cox proportional hazards model identified TACE with a 2.765-fold increased risk of mortality compared with SR (95% confidence interval: 1.853–4.127, *p*<0.001).

**Conclusions:**

Patients with solitary large HCC should be classified at least as intermediate stage HCC. SR provides significantly better survival than TACE for solitary large HCC regardless of tumor stage. Further amendment to the BCLC classification is mandatory.

## Introduction

Liver cancer accounts for more than 700,000 deaths each year, and is a major cause of cancer-related deaths globally.[1] Screening for hepatocellular carcinoma (HCC) in high-risk individuals to detect tumors at a curable stage is recommended by the current American Association for the Study of Liver Diseases (AASLD) and European Association for the Study of Liver (EASL) management guidelines.[2, 3] Despite increased detection rate of small HCC by the screening programs, large HCC at disease presentation still remains common. Up to 32% of HCC had a tumor diameter > 5 cm, and another 10–20% of tumors were larger than 10 cm at the time of diagnosis.[4, 5]

Solitary large (single tumor > 5 cm in diameter) HCC presents a unique challenge to clinicians. The optimal classification and management for patients with solitary large HCC is under heated and ongoing debate. Current HCC management guidelines recommend curative treatment including surgical resection (SR) and radiofrequency ablation (RFA) for early stage HCC, whereas transarterial chemoembolization (TACE) is offered to patients with intermediate stage tumor.[2, 3, 6] The Barcelona Clinic Liver Cancer (BCLC) classification is the most widely used staging system and is endorsed by the AASLD and EASL guidelines.[2, 3] In the original BCLC classification, resectability, rather than tumor size, was emphasized as an indicator between early and intermediate stage HCC.[7] However, other reports advocate 5 cm as the cut-off point between early and intermediate stage HCC (Table 1). The lack of clarity and resultant incongruity in interpretation of HCC staging continued to date.[8, 9]

**Table 1:** Comparison of definitions on early and intermediate hepatocellular carcinoma in current literatures.

Authors	Year	Source	Early HCC	Intermediate HCC	Remark
Llovet et al.[7]	1999	Table	Single, 3 tumors <3cm	Large/Multinodular	Original BCLC system
Bruix et al.[34]	2001	Text	Single <5cm, 3 tumors <3cm	Not qualify for curative options	EASL conference
Bruix et al.[35]	2002	Text	Single ≤5cm, 3 tumors ≤3cm	Exceeding stage A	
		Figure	Single, 3 tumors ≤3cm	Multinodular	
Llovet et al.[36]	2003	Text	Single <5cm, 3 tumors <3cm	Exceeding stage A	
		Figure	Single, 3 tumors <3cm	Multinodular	
Bruix et al.[37]	2005	Text	Single, 3 tumors ≤3cm	Large/Multinodular	AASLD guideline
Forner et al.[38]	2010	Text	Single, 3 tumors <3cm	Single large HCC, Multinodular	BCLC system update
Omata et al.[24]	2010	Figure	Single ≤5cm, 3 tumors ≤3cm	Single>5cm, >3tumors	APASL guideline
Bruix et al.[2]	2011	Text	Single, 3 tumors ≤3cm	Large/Multinodular	AASLD guideline
EASL-EORTC[3]	2012	Text	Single, 3 tumors <3cm	Multinodular	EASL-EORTC guideline
		Figure	Single, 3 tumors ≤3cm	Multinodular	
Bolondi et al.[39]	2012	Text& Figure	Single resectable tumor 3 tumors <3cm	Single unresectable tumor >5cm Multinodular >3cm	Proposal of B1-B4 subclassification
Verslype et al.[25]	2012	Figure	Single <5cm, 3 tumors <3cm	Multinodular	ESMO-ESDO guideline
Bolondi et al.[26]	2013	Text	Single <5cm, 3 tumors <3cm		AISF statement
Méndez-Sánchez et al.[27]	2014	Text	Single ≤5cm, 3 tumors ≤3cm	Large/Multinodular	LAASL guideline

Patients with single tumor ≤2cm and patients with portal vein tumor thrombosis, extrahepatic tumor spreading, suboptimal performance status and Child-Turcotte-Pugh classification C cirrhosis are not included in this table.

AASLD, American Association for the Study of Liver Diseases; AISF, Italian Association for the Study of the Liver; APASL, Asian Pacific Association for the Study of the Liver; BCLC, Barcelona Clinic Liver Cancer; EASL, European Association for the Study of the Liver; EORTC, European Organisation for Research and Treatment of Cancer; ESDO, European Society of Digestive Oncology; ESMO, European Society for Medical Oncology; HCC, hepatocellular carcinoma, LAASL, Latin American Association for the Study of the Liver.

For patients with solitary large HCC, the role of RFA is limited due to smaller thermal ablation zone of 3–4 cm with conventional RFA devices.[10] SR has been advocated as a safe procedure with reasonable long-term prognosis.[11, 12] The efficacy of TACE in patients with large HCC has also been reported.[13] However, the comparative efficacy between SR and TACE in solitary large HCC remains to be determined. This study has two aims. First, by analyzing a large treated cohort of HCC patients with preserved liver function, we determined whether solitary large HCC is better fitted into early or intermediate stage HCC. Second, we investigated the survival of patients with solitary large HCC regardless of tumor stage undergoing SR or TACE as their primary anti-cancer therapy. A propensity score matching analysis was used to reduce potential bias and to compare the long-term outcomes between patients with solitary large HCC treated by SR or TACE.

## Patients and Methods

### Patients

A total of 3,117 patients with newly diagnosed HCC admitted to Taipei Veterans General Hospital were retrospectively reviewed. Patients with early or intermediate stage HCC as well as patients with solitary large (> 5 cm) HCC regardless of BCLC stages were enrolled. Comprehensive demographics, tumoral status and liver disease burden were documented when diagnosis was confirmed. The long-term survival of patients was recorded. The study complies with the Declaration of Helsinki and had been approved by the Institutional Review Board of Taipei Veterans General Hospital (IRB protocol number 2014-06-013AC).

### Diagnosis and Treatments

The diagnosis of HCC was confirmed based on HCC management guidelines from EASL or AASLD.[2, 3] Performance status was determined when HCC diagnosis was made.[14] Total tumor volume (TTV) was calculated based on tumor diameter.[15] Patients were censored at time of liver transplantation if they received transplantation as salvage therapy.

To investigate the impact of tumor nodularity and size on treatment allocation and long-term prognosis in patients without portal vein tumor thrombosis (PVTT) or tumor-related symptoms and with adequate liver function reserve (fulfilling either BCLC stage A or stage B), we defined three groups of patients. Group A was defined as patients having solitary tumor ranging from 2 cm to 5 cm in size or no more than 3 tumors not exceeding 3 cm in diameter. Group B encompassed patients with multiple tumors beyond 3 cm. Finally, group SL were patients with single tumor larger than 5cm in size. Group A and group B were patients who were unambiguously classified as BCLC stage A or stage B by all experts. Group SL was isolated in order to examine the survival of patients with solitary large HCC.

The eligibility criteria of SR and TACE were reported previously.[16, 17] In general, SR was considered in patients with no main portal trunk involvement.[18] TACE was directed to patients without obvious contraindications to TACE or to patients unwilling to undergo SR.[19] TACE was also performed under emergent condition for patients with acute tumor hemorrhage. Full written informed consent was obtained before each treatment procedure, and patient records/information was anonymized and de-identified prior to analysis for this retrospective study.

### Propensity score matching analysis

We employed a propensity score matching analysis to investigate the relation between treatment modalities and clinical outcomes in this observational, non-randomized study. The propensity score method reduced bias in patient selection and generated matched pairs of patients for comparison of the long-term outcomes between SR and TACE.[20, 21] Variables which might affect the treatment selection process were comprehensively included in the propensity score model. Binary logistic regression was employed to determine a continuous propensity score from 0 to 1. A nearest-neighbor match without replacement in a one-to-one ratio was performed to select patients receiving SR and TACE into further investigations. A pre-defined caliper width (0.2 of the standard deviation of the logit of the propensity score) was selected for better performance in estimating treatment effects.[22]

### Statistical analysis

The two-tailed Fisher exact test or the Chi-squared test was employed for categorical variables. The Kruskal-Wallis test or the Mann-Whitney *U* test was utilized to compare continuous variables between two or more patient groups. Survival analysis was performed by the Kaplan-Meier method. Univariate analysis was used to identify potential prognostic factors. Variables with *p* value less than 0.1 in the univariate analysis were introduced into the Cox proportional hazards model where the adjusted hazard ratios (HR) and 95% confidence intervals (CI) were determined. A final *p* value less than 0.05 was considered significant. The statistical analysis was performed with SPSS for Windows version 21 (IBM, NY, USA).

## Results

### Characteristics and survival of patients with early and intermediate HCC

**Fig 1:**
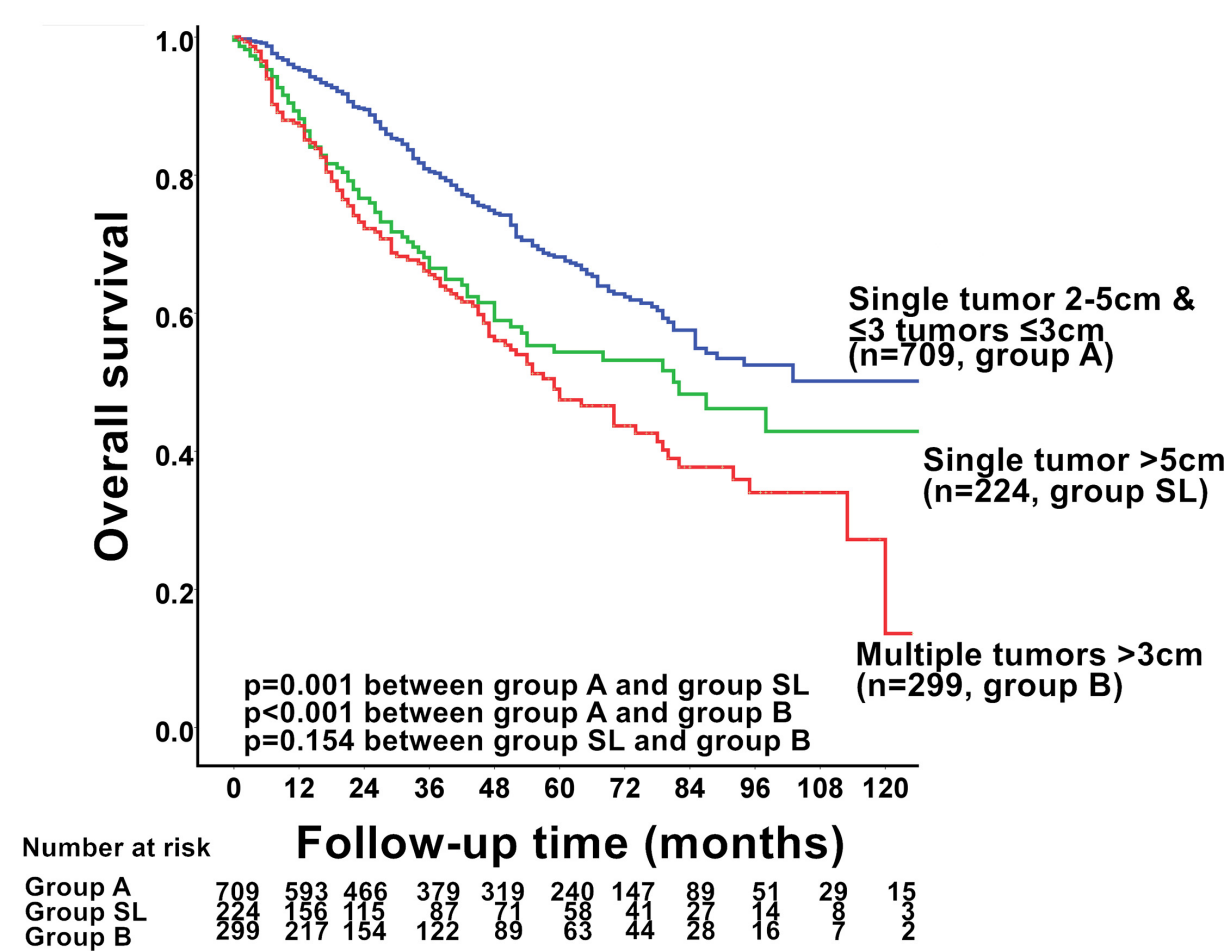
Comparison of survival between hepatocellular carcinoma (HCC) patients with single tumor ranging from 2–5cm or up to 3 tumors ≤ 3 cm (group A), single tumor > 5 cm (group SL), and multiple tumors > 3 cm (group B). Group A patients had significantly better long-term survival than group SL and group B patients (*p* = 0.001 and *p*<0.001, respectively). The prognosis was similar between group SL and group B patients (*p* = 0.154).

A total of 1,232 (40%) newly diagnosed HCC patients met the criteria for either BCLC stage A or stage B. Among these patients, 709 (58%) patients were classified as group A, whereas 224 (18%) and 299 (24%) patients were categorized as group SL and group B, respectively (Table 2). There were significantly different distributions on etiologies of chronic hepatic diseases, serum biochemistries, α-fetoprotein (AFP) level, coagulation function, and tumor burden among group A, group SL, and group B patients (*p*<0.05). The three groups received different anti-cancer treatments (*p*<0.05). Group A patients had significantly better long-term survival when compared with group SL or group B patients (*p* = 0.001 and *p*<0.001, respectively; Fig 1). The long-term prognosis between group B and group SL were similar (*p* = 0.154). Among group A HCC, patients with single tumor 2–5 cm had similar long-term survival compared with patients with up to 3 tumors no larger than 3 cm (*p* = 0.166).

**Table 2:** Baseline demographics between HCC patients stratified by tumor number and size.

Variables	Single 2–5cm; 3 tumors ≤ 3cm (n = 709, group A)	Single > 5cm (n = 224, group SL)	Multiple tumors > 3cm (n = 299, group B)	*p* value
Age (years, mean ± SD)	65±12	63±15	64±12	0.755
Male, n (%)	509(72)	194(87)	238(80)	<0.001
Positive for HBsAg, n (%)	365(52)	133(59)	172(58)	0.054
Positive for anti-HCV, n (%)	281(40)	33(15)	96(32)	<0.001
Alcoholism, n (%)	90(13)	28(12)	42(14)	0.820
Serum biochemistry (mean ± SD)				
Albumin (g/dL)	3.9±0.5	3.9±0.5	3.8±0.5	0.042
Bilirubin (mg/dL)	1.0±0.7	0.9±1.1	0.9±0.6	0.024
Creatinine (mg/dL)	1.1±1.0	1.2±0.8	1.2±1.0	0.010
INR of PT	1.1±0.1	1.0±0.1	1.0±0.1	<0.001
ALT (U/L)	65±59	57±58	67±65	0.003
Sodium (mmol/L)	140±3	139±3	140±3	0.195
AFP (ng/mL, mean ± SD)	744±10144	10366±42923	18856±250913	0.001
CTP class A/B (%)	88/12	92/8	90/10	0.174
CTP score (mean ± SD)	5.5±0.8	5.4±0.7	5.5±0.8	0.374
Number of tumor (s) 1/2/≥3 (%)	73/20/7	100/0/0	0/43/57	<0.001
Total tumor volume (cm^3^, mean ± SD)	17±15	484±562	250±519	<0.001
Portal vein tumor thrombosis, n (%)	0 (0)	0 (0)	0 (0)	1.000
Performance status 0 (%)	709 (100)	224 (100)	299 (100)	1.000
TIS 0/1/2/3/4/5/6 (%)	73/25/2/0/0/0/0	0/39/21/23/15/2/0	31/35/16/11/7/0/0	<0.001
CLIP 0/1/2/3/4/5/6 (%)	54/40/6/0/0/0/0	49/17/18/14/2/0/0	0/61/30/9/0/0/0	<0.001
Treatment(SR/RFA/TACE/Other)	41/31/23/5	56/3/34/7	26/7/57/10	<0.001

AFP, α-fetoprotein; ALT, alanine transaminase; BCLC, Barcelona Clinic Liver Cancer; CLIP, Cancer of the Liver Italian Program; CTP, Child-Turcotte-Pugh; HBsAg, hepatitis B surface antigen; HCC, hepatocellular carcinoma; HCV, hepatitis C; INR, international normalized ratio; PT, prothrombin time; RFA, radiofrequency ablation; SD, standard deviation; SR, surgical resection; TACE, transarterial chemoembolization; TIS, Taipei Integrated Scoring System

During a median follow-up duration of 33 months, 194 (27%), 76 (34%) and 123 (41%) of patients of group A, group SL, and group B, respectively, died. The estimated 1-, 3-, and 5-year survival rates in group A, group SL, and group B were 96% *vs*.90% *vs*. 88%, 81% *vs*. 68% *vs*. 66%, and 68% *vs*. 54% *vs*. 49%, respectively.

### Patients with solitary large HCC regardless of BCLC stages

A total of 803 (26%) newly diagnosed HCC patients with solitary tumor larger than 5 cm in size were identified during study period. Among them, 240 (30%) and 229 (29%) patients received SR and TACE, respectively, as the primary therapy. The propensity score matching analysis identified 156 matched pairs of patients from each treatment group to compare therapeutic efficacy and survival.

### Characteristics and survival of patients with solitary large HCC regardless of BCLC stages receiving SR or TACE

**Fig 2:**
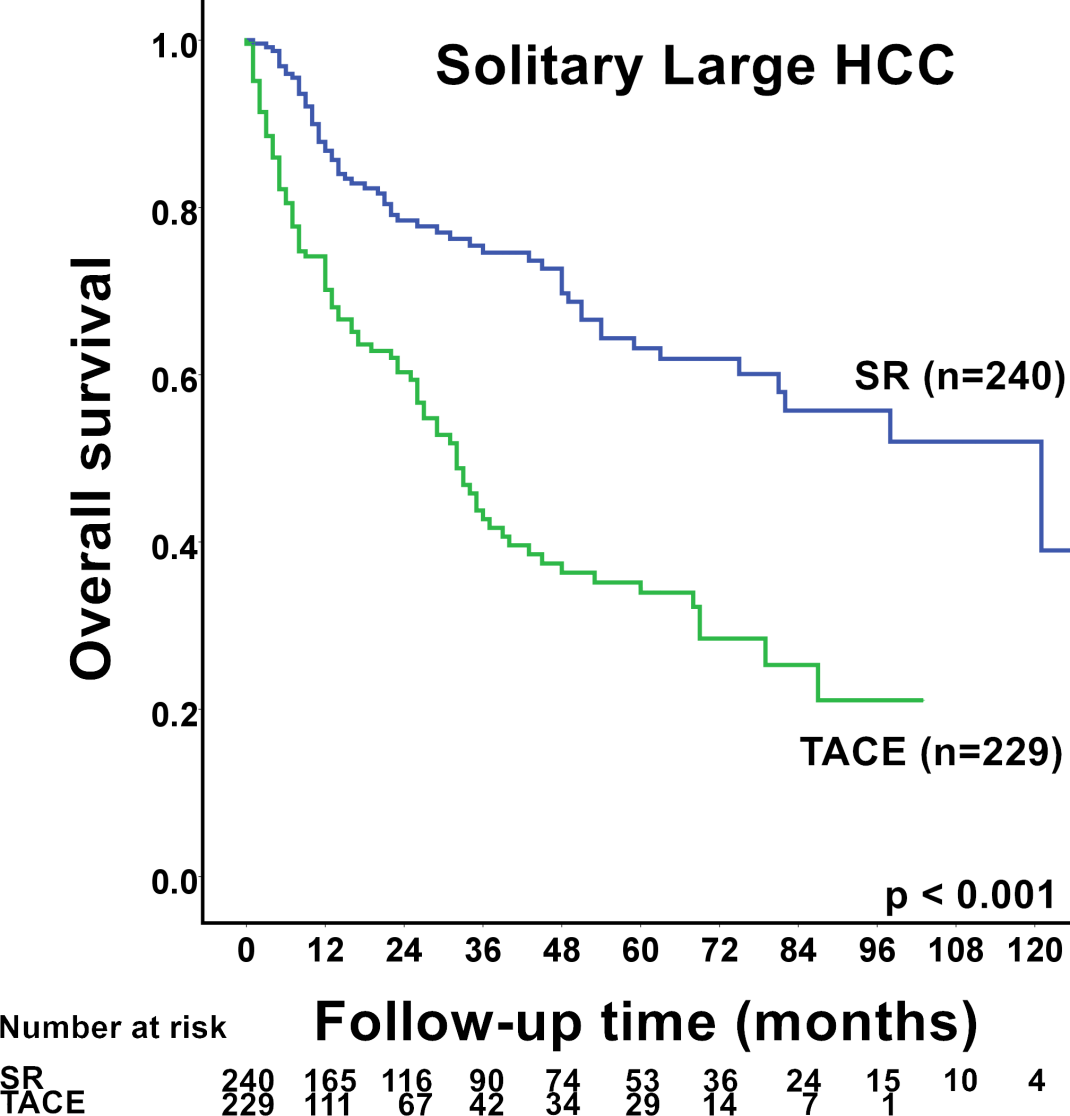
Comparison of survival between solitary large (> 5 cm) hepatocellular carcinoma (HCC) patients receiving surgical resection (SR) or transarterial chemoembolization (TACE). Solitary large HCC patients receiving SR had significantly better long-term survival than patients receiving TACE (*p*<0.001).

Significantly diverse etiologies of hepatic diseases were found in patients with solitary large HCC undergoing SR or TACE (*p*<0.05; Table 3). The TACE group was significantly older and had more severe liver cirrhosis (both *p*<0.001). Alternately, the SR group was associated with fewer portal vein thrombosis, smaller TTV, lower TIS and CLIP score, and better performance status (all *p*<0.05). Solitary large HCC patients receiving SR had statistically significantly improved survival compared to patients undergoing TACE (*p*<0.001; Fig 2). During the follow-up duration, 63 (26%) and 101 (44%) of patients who underwent SR and TACE died, respectively. The 1-, 3-, and 5-year survival rates in the SR and TACE group were 88% *vs*. 74%, 76% *vs*. 44% and 63% *vs*. 35%, respectively (*p*<0.001).

**Table 3:** Baseline demographics in solitary large hepatocellular carcinoma patients receiving SR or TACE.

Variables	SR (n = 240)	TACE (n = 229)	*p* value
Age (years, mean ± SD)	59±14	68±14	<0.001
Male, n (%)	199 (82)	192 (84)	0.805
Positive for HBsAg, n (%)	155 (65)	114 (50)	0.001
Positive for anti-HCV, n (%)	34 (14)	52 (23)	0.017
Alcoholism, n (%)	34 (14)	38 (17)	0.522
Serum biochemistry (mean ± SD)			
Albumin (g/dL)	4.0±0.5	3.7±0.6	<0.001
Bilirubin (mg/dL)	0.9±1.0	1.1±1.2	0.002
Creatinine (mg/dL)	1.1±0.9	1.2±1.1	0.140
INR of PT	1.0±0.1	1.1±0.1	0.034
ALT (U/L)	73±146	75±99	0.060
Sodium (mmol/L)	139±3	138±4	0.001
AFP (ng/mL, mean ± SD)	15161±56942	29192±166805	0.624
Performance status 0/1/2-4 (%)	64/29/7	51/23/26	<0.001
CTP class A/B/C (%)	92/8/0	80/17/3	<0.001
CTP score (mean ± SD)	5.3±0.7	5.9±1.3	<0.001
MELD score (mean ± SD)	8.3±2.5	9.3±3.3	<0.001
Number of tumor (s) 1/2/≥3 (%)	100/0/0	100/0/0	1.000
Total tumor volume (cm^3^, mean ± SD)	567±690	751±1118	0.029
Portal vein tumor thrombosis, n (%)	46(20)	82(36)	<0.001
TIS 0/1/2/3/4/5 (%)	0/33/24/22/19/2/0	0/22/22/30/20/5/1	0.031
CLIP 0/1/2/3/4/5%)	38/20/18/14/10/0/0	23/21/23/17/12/3/1	0.002

AFP, α-fetoprotein; ALT, alanine transaminase; CLIP, Cancer of the Liver Italian Program; CTP, Child-Turcotte-Pugh; HBsAg, hepatitis B surface antigen; HCV, hepatitis C; INR, international normalized ratio; MELD, model for end-stage liver disease; PT, prothrombin time; SD, standard deviation; SR, surgical resection; TACE, transarterial chemoembolization; TIS, Taipei Integrated Scoring System

### Characteristics and survival of patients with solitary large HCC regardless of BCLC stages receiving SR or TACE in the propensity model

**Fig 3:**
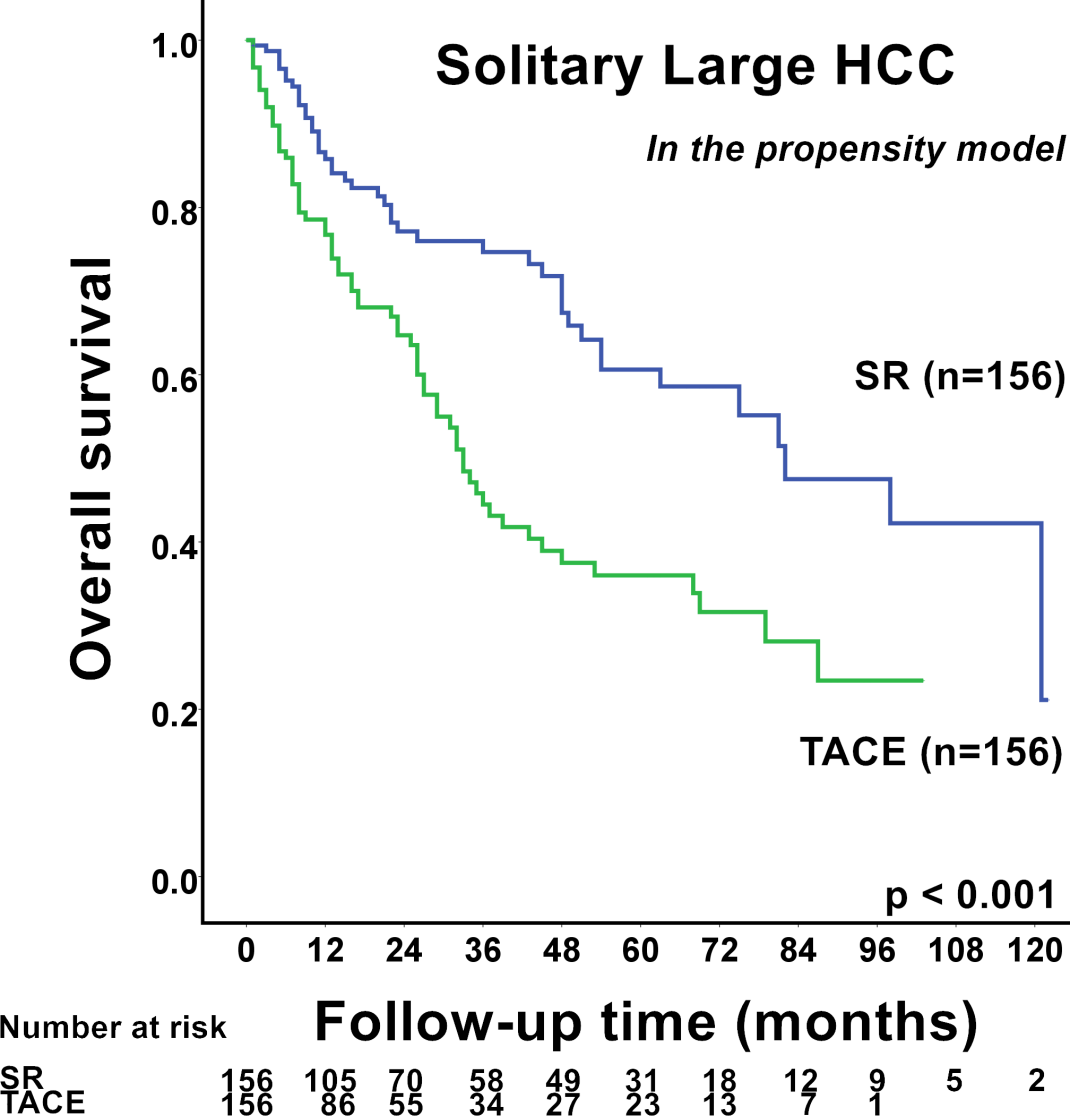
Comparison of survival between solitary large (> 5 cm) hepatocellular carcinoma (HCC) patients receiving surgical resection (SR) or transarterial chemoembolization (TACE) in the propensity model. Patients with solitary large HCC receiving SR had significantly better long-term survival than patients receiving TACE in the propensity model (*p*<0.001).

Table 4 shows the characteristics of HCC patients between the SR or TACE group in the propensity model. The baseline demographics, including etiologies of underlying hepatic diseases, serum biochemistry, degree of cirrhosis, tumoral factors, CLIP score, and performance status, were similar between SR and TACE group in the propensity model. SR patients in the propensity model had better long-term survival compared with TACE patients (*p*<0.001; Fig 3). During the follow-up period, 45 (29%) and 69 (44%) patients in the SR and TACE group, respectively, died. The 1-, 3-, and 5-year survival rates of patients receiving SR and TACE were 87% *vs*. 79%, 76% *vs*. 46% and 61% *vs*. 36% (*p*<0.001). Compared with TACE patients, SR patients had significantly better prognosis even when patients with PVTT were excluded from the analysis (*p*<0.001). SR was associated with improved survival both when tumor size was 5.0–9.9 cm (*p* = 0.017) and when tumor size ≥ 10.0 cm (*p*<0.001).

**Table 4:** Baseline demographics in solitary large hepatocellular carcinoma patients receiving SR or TACE in the propensity model.

Variables	SR (n = 156)	TACE (n = 156)	*p* value
Age (years, mean ± SD)	64±13	66±15	0.350
Male, n (%)	131 (84)	133 (85)	0.875
Positive for HBsAg, n (%)	91 (58)	83 (53)	0.425
Positive for anti-HCV, n (%)	27 (17)	28 (18)	1.000
Alcoholism, n (%)	20 (13)	24 (15)	0.626
Serum biochemistry (mean ± SD)			
Albumin (g/dL)	3.9±0.5	3.9±0.4	0.443
Bilirubin (mg/dL)	0.9±0.7	0.9±0.5	0.072
Creatinine (mg/dL)	1.1±0.6	1.1±0.8	0.879
INR of PT	1.0±0.1	1.0±0.1	0.785
ALT (U/L)	71±102	71±84	0.170
Sodium (mmol/L)	139±3	139±4	0.633
AFP (ng/mL, mean ± SD)	16172±62893	22097±88044	0.787
Performance status 0/1/2-4 (%)	59/32/9	63/22/15	0.079
CTP class A/B (%)	89/11	92/8	0.436
CTP score (mean ± SD)	5.4±0.7	5.4±0.8	0.923
MELD score (mean ± SD)	8.5±2.7	8.7±2.4	0.305
Number of tumor (s) 1/2/≥3 (%)	100/0/0	100/0/0	1.000
Total tumor volume (cm^3^, mean ± SD)	609±755	693±930	0.365
Intrahepatic/extrahepatic portal vein tumor thrombosis, n (%)	34/5 (22/3)	31/12 (20/8)	0.213
TIS 0/1/2/3/4/5 (%)	0/31/24/19/22/4/0	0/28/22/31/16/3/0	0.163
CLIP 0/1/2/3/4/5 (%)	37/18/17/13/15/0/0	31/21/23/15/8/2/0	0.146

AFP, α-fetoprotein; ALT, alanine transaminase; CLIP, Cancer of the Liver Italian Program; CTP, Child-Turcotte-Pugh; HBsAg, hepatitis B surface antigen; HCV, hepatitis C; INR, international normalized ratio; MELD, model for end-stage liver disease; PT, prothrombin time; SD, standard deviation; SR, surgical resection; TACE, transarterial chemoembolization; TIS, Taipei Integrated Scoring System

### Prognostic factors analysis for solitary large HCC

The univariate survival analysis revealed the following predictors of mortality in patients with solitary large HCC: serum albumin < 4 g/dL, bilirubin ≥ 1 mg/dL, α-fetoprotein (AFP) level ≥ 400 ng/mL, performance status ≥ 1, tumor size > 10 cm, presence of PVTT, and TACE treatment (all *p*<0.01, Table 5). In the adjusted Cox multivariate survival analysis, there were 5 independent predictors associated with adverse outcome: serum albumin < 4 g/dL (HR 1.621, 95% CI 1.175–2.236, *p* = 0.003), AFP level ≥ 400 ng/mL (HR 2.223, 95% CI 1.591–3.107, *p*<0.001), tumor size > 10 cm (HR 1.501, 95% CI 1.081–2.083, *p* = 0.015), presence of PVTT (HR 1.919, 95% CI 1.385–2.658, *p*<0.001) and TACE (HR 2.922, 95% CI 2.090–4.086, *p*<0.001).

**Table 5:** Univariate and multivariate survival analysis in solitary large hepatocellular carcinoma patients undergoing SR or TACE.

	Number	Univariate analysis				Multivariate analysis		
		HR	CI	*p*		HR	CI	*p*
All patients with solitary large hepatocellular carcinoma (n = 469)								
Age (<65/≥65 years)	235/234	0.932	0.685–1.266	0.651				
Sex (male/female)	391/78	1.065	0.704–1.610	0.766				
Albumin (≥4.0/<4.0g/dL)	206/263	1.822	1.328–2.500	<0.001		1.621	1.175–2.236	0.003
Bilirubin (<1/≥1mg/dL)	320/149	1.666	1.215–2.283	0.002				
INR of PT (<1/≥1)	172/297	1.271	0.925–1.746	0.139				
Creatinine (<1/≥1mg/dL)	236/233	0.934	0.687–1.269	0.663				
Sodium (≥140/<140 mmol/L)	214/255	1.269	0.932–1.729	0.130				
AFP (<400/≥400ng/mL)	298/171	1.903	1.389–2.606	<0.001		2.223	1.591–3.107	<0.001
Performance status (0/≥1)	270/199	1.596	1.163–2.191	0.004				
Tumor size (≤10/>10cm)	305/164	1.790	1.306–2.453	<0.001		1.501	1.081–2.083	0.015
Portal vein tumor thrombosis (no/yes)	341/128	2.410	1.749–3.321	<0.001		1.919	1.385–2.658	<0.001
Treatment (SR/TACE)	240/229	2.639	1.917–3.633	<0.001		2.922	2.090–4.086	<0.001
Patients selected in the propensity model (n = 312)								
Age (<65/≥65 years)	142/170	0.653	0.451–0.947	0.024				
Sex (male/female)	264/48	0.959	0.564–1.632	0.877				
Albumin (≥4.0/<4.0g/dL)	140/172	1.517	1.044–2.204	0.029				
Bilirubin (<1/≥1mg/dL)	219/93	1.471	0.998–2.169	0.051				
INR of PT (<1/≥1)	121/191	1.131	0.776–1.648	0.522				
Creatinine (<1/≥1mg/dL)	150/162	0.802	0.555–1.159	0.240				
Sodium (≥140/<140 mmol/L)	149/163	1.251	0.863–1.813	0.238				
AFP (<400/≥400ng/mL)	200/112	1.989	1.356–2.918	<0.001		2.163	1.441–3.247	<0.001
Performance status (0/≥1)	191/121	1.237	0.833–1.836	0.292				
Tumor size (≤10/>10cm)	206/106	1.785	1.223–2.604	0.003		1.585	1.066–2.356	0.023
Portal vein tumor thrombosis (no/yes)	230/82	2.168	1.467–3.203	<0.001		1.901	1.271–2.843	0.002
Treatment (SR/TACE)	156/156	2.118	1.448–3.099	<0.001		2.765	1.853–4.127	<0.001

The forepart of the parentheses was set as the reference group in the univariate and multivariate analysis

AFP, α-fetoprotein; CI, confidence interval; HR, hazard ratio; INR, international normalized ratio; PT, prothrombin time; SR, surgical resection; TACE, transarterial chemoembolization

Univariate survival analysis for patients in the propensity score model showed the following predictors of poor prognosis: age < 65 years, serum albumin < 4 g/dL, bilirubin ≥ 1 mg/dL, AFP level ≥ 400 ng/mL, tumor size > 10 cm, presence of PVTT, and TACE treatment (all *p*<0.1). In the adjusted Cox multivariate survival analysis, we confirmed 4 independent factors linked with decreased survival: AFP level ≥ 400 ng/mL (HR 2.163, 95% CI 1.441–3.247, *p*<0.001), tumor size > 10 cm (HR 1.585, 95% CI 1.066–2.356, *p* = 0.023), PVTT (HR 1.901, 95% CI 1.271–2.843, *p* = 0.002) and TACE treatment (HR 2.765, 95% CI 1.853–4.127, *p*<0.001).

## Discussion

There are intense debates on the classification and management of patients with solitary large (> 5 cm) HCC. We evaluated a large cohort of patients with solitary large HCC to investigate the impact of tumor size on HCC stratification and treatment strategies. From prognostic perspective, we demonstrate that patients with solitary large HCC with preserved hepatic function and without PVTT or tumor-related symptoms should more appropriately fit into intermediate stage. We also show that for patients with solitary large HCC regardless of liver reserve, status of portal vein invasion and performance status, SR was associated with a significantly better long-term prognosis compared with TACE. Moreover, the Cox multivariate analysis confirmed that TACE was independently linked with decreased survival. Our results provide solid evidence for cancer staging and treatment in patients with solitary large HCC.

Traditionally, the survival data of HCC patients are based on untreated patients. With the advances in cancer management, the only possible approach to define prognosis and staging is to review the outcomes after treatment.[7] We demonstrated that there was no significant difference in long-term survival between patients with single HCC ≥ 5 cm and patients with multiple HCCs larger than 3 cm. Therefore patients with solitary large HCC should be classified as intermediate stage to better represent their nature and prognosis of disease. This staging strategy is not uncommon in current clinical practice. In a large cohort from the HCC East-West study group reporting outcomes after SR, patients with solitary large HCC were classified as intermediate stage.[23] Furthermore, several consensus guidelines from Asia-Pacific, Europe, Latin America, and Italy all limit early stage HCC to those with single tumor ≤ 5 cm and up to 3 tumors ≤ 3 cm.[24–27]

The resectability has been emphasized by the BCLC group as the boundary between early and intermediate stage HCC in patients with solitary large tumor.[28] The BCLC group stated that single tumor beyond 5 cm should still be considered for surgical resection; in addition, patients with resectable solitary large HCC should be classified as early stage HCC, and unresectable solitary large HCC would be considered intermediate stage HCC.[3, 28, 29] However, a recent study using regret-based decision curve analysis to assess physician preferences toward SR and TACE on intermediate stage HCC showed a significant separation among physicians’ preferences.[30] Therefore, using resectability *per se* as the staging criterion may raise uncertainty and add difficulties in comparing results between different institutions. Notably, the eligibility of patients to receive RFA, TACE or targeted therapy was not included in the staging criteria of early, intermediate and advanced stage HCC. Taken together, we propose that patients with single HCC larger than 5 cm with no tumor-related symptoms, no PVTT, and with preserved liver function should be classified as intermediate stage HCC regardless of the treatment they receive.

The management of solitary large HCC remains a major treatment challenge. According to the current BCLC scheme, TACE is the recommended treatment for patients with intermediate stage HCC. TACE has been shown safe and effective in treating larger HCC.[13] However, therapeutic modalities including RFA or TACE are still potentially limited by a lack of complete tumor eradication.[4] Significant progress has been achieved in patient selection, surgical techniques, and post-operative management of HCC in recent years, and SR was associated with improved outcomes in carefully selected solitary large HCC patients.[5] To further clarify the impact of treatment selection on long-term prognosis, we analyzed a large cohort with solitary large HCC irrespective of their performance status, hepatic functional reserve or PVTT. We demonstrate that for patients with solitary large HCC, SR was associated with a better overall survival compared with TACE.

Since patients undergoing SR are usually highly selected, we utilized propensity score matching analysis to minimize the confounding effect of treatment allocation in this non-randomized, retrospective study. In the propensity model, patients in the SR or TACE group were well matched in baseline characteristics, hepatic functional reserve and performance status. We found that the SR group had better prognosis than TACE group in the propensity model. Consistently, in the Cox multivariate model, TACE was confirmed a significant predictor associated with poor long-term survival compared with SR after adjusting for confounders in all-patient group and in patients selected in the propensity model. SR should be thus considered a priority treatment for patients with solitary large HCC.

Consistent with reports from other study groups, this study demonstrates the applicability of SR in patients with solitary large HCC irrespective of their performance status, liver reserve or presence of PVTT.[31] Moreover, we also suggest that patients with solitary large HCC should be classified at least as intermediate stage HCC, for which the recommended treatment would be TACE. These seemingly contradictory statements encourage clinicians to individualize HCC management to obtain better treatment results. The BCLC system has been acknowledged to stratify patients to distinct stages that subsequently link to specific therapies.[32] However, due to high heterogeneity among HCC patients especially at the intermediate stage, further sub-classification and refinement of the BCLC staging system is urgently needed.[33]

This study has a few limitations. First, the retrospective nature may make selection bias unavoidable even in the propensity score model. Although the baseline demographics were similar between treatment groups, there may be hidden characteristics that cannot be directly compared. Second, HCC is a highly heterogeneous cancer, and treatment allocation is partly dependent on physician’s preference. Third, certain co-morbid illnesses, including chronic heart or pulmonary diseases, were not included in the analysis. Last, the results of this study are from a single tertiary referral center, therefore external validation is needed from different sources.

In conclusion, our results suggest that patients with solitary large HCC should be classified at least as intermediate stage HCC. With improvement in patient selection and treatment strategy, solitary large HCC is not a contraindication to aggressive therapy. Further amendment to the BCLC scheme is urgently required. We confirm that SR offers better long-term survival than TACE in patients with solitary large HCC. Our data provide evidenced-based approach for cancer staging and treatment for this special patient group.

## Abbreviations

AASLD: American Association for the Study of Liver Diseases
AFP: α-fetoprotein
AISF: Italian Association for the Study of the Liver
ALT: alanine transaminase
APASL: Asian Pacific Association for the Study of the Liver
BCLC: Barcelona Clinic Liver Cancer
CI: confidence interval
CLIP: Cancer of the Liver Italian Program
CTP: Child-Turcotte-Pugh
EASL: European Association for the Study of the Liver
EORTC: European Organisation for Research and Treatment of Cancer
ESDO: European Society of Digestive Oncology
ESMO: European Society for Medical Oncology
HBsAg: hepatitis B surface antigen
HCC: hepatocellular carcinoma
HCV: hepatitis C virus
HR: hazard ratio
INR: international normalized ratio
LAASL: Latin American Association for the Study of the Liver
MELD: model for end-stage liver disease
PT: prothrombin time
PVTT: portal vein tumor thrombosis
RFA: radiofrequency ablation
RR: relative risk
SD: standard deviation
SR: surgical resection
TACE: transarterial chemoembolization
TIS: Taipei Integrated Scoring System
TTV: total tumor volume
